# Appeals

**Published:** 1889-12-21

**Authors:** 


### FALLOW CROPS.

### APPEALS.

This is the season of appeals. For weeks past our charitable institutions have been severally engaged in preparation for the great annual effort to replenish their fast-emptying treasuries, and now flood the post with their apparently insatiable demands. The people—comparatively few—whose names appear as donors in the various lists are the targets at which every thrilling application is directed. Givers once, they must remain givers always, and well may they stand dismayed at the prospect. At the same time their neighbours are left severely alone or at most suffer the trifling inconvenience of an occasional petition sent by a blundering hand, and which, as it never finds its way to their hearts, they have no difficulty in ignoring.

This, too, is the period when a few gentlemen, presumably of ample leisure and small patience, write to the newspapers complaining that their time should be wasted in committing appeals to the flames or the waste-paper basket. Correspondents of this sort invariably intimate that the issue of an appeal is unnecessary and even prejudicial to the cause advocated, and that they have decided never to give to any-one who asks, because—chaste conception of an innocent but ruffled mind!—they are persuaded that a really useful work will never need any advocacy but its own merit. Nothing could well be more fallacious. Doubtless, a good work will often achieve a reputation, and even secure genuine admiration; but, alas! not so often does this sentiment develop into an effort to assist it. As regards so-called public charities, they are not seldom in the position of a beautiful flower, to which many are attracted ; yet the disposition is not to nurture the plant, but to pluck the blossom. Similarly, the appreciation of a large number of well-intentioned folk bestowed upon a charitable institution is oftenest illustrated by a desire to share in the fruits.

Quite recently, a querulous letter anent appeals addressed to a daily paper drew forth a reply, where it was stated that a particular hospital whence no appeals had emanated for two or three months, and which meanwhile had incurred an expenditure of some thousands of pounds, had received during that period less than £70 in voluntary contributions. And we fear this is no case of exceptional misfortune, but a fair illustration of the conditions under which many a work of public utility is administered. Whatever the causes, and the bewildering multiplicity of societies is undoubtedly one, charity lacks spontaneity, and unless the needs of an institution are pressed home they are certain to be overlooked.

As a logical consequence, but one greatly to be deplored, experience shows that the cause itself counts less towards success than the way in which it is represented. It is possible for very insignificant "institutions" to attract to themselves funds while better undertakings languish. Whatever amateur critics may say, plain substantial merit seldom appeals successfully to the palate. This is an age of condiments, and not even the charitable appetite is able to dispense with them. It ought to be made obligatory upon all begging charities to set forth with each appeal a certified statement of the ingredients employed in its manufacture—how much truth and how much fiction. This would resolve itself into a plain certificate of the work actually accomplished by the organisation appealing. Hospitals ought to state the number of beds maintained and the total annual expenditure, with other details. Of course, a small society may be well deserving of support, but in no case ought it to be possible for that support to be given in the belief that the institution is a large one. As it is, not a few small and even baneful institutions deliver themselves in verses of deluding magnitude, and many a donor who has been persuaded by specious appeals into generous benefactions to unknown societies has not only failed to advantage the cause of real charity, but has afterwards learned to regard with suspicion the petitions of unquestionable undertakings. Genuine medical institutions would be better protected if all applications for aid were required to bear the names of the medical and surgical staff; better still, if hospitals before soliciting public assistance were compelled to take out some sort of license.

If, as we have tried to show, appeals are indispensable, we do not love them any the more on that account, and those who object to receiving them may rest assured that managers would only be too thankful to be relieved of the necessity for their issue. Think of the time, energy, and hard cash expended upon them—too often to secure a very inadequate result. No better illustration of the heart-breaking difficulties in the way of raising money for charitable purposes could there be than the record of a meeting in aid of a meritorious object held a year or more ago at the Mansion House, with the Lord Mayor in the chair, when a Royal Princess was present, and a powerful address was delivered by a Cabinet Minister, supported by other eminent personages. The result was a collection of £6 5s. 6d. The expenses were not stated.

An appeal which has taken months to prepare may fail altogether if it should be issued at a time when minds or purses are preoccupied, and the slightest check or hindrance may be fatal to success. A colliery disaster at home or a famine abroad means direct loss to the established institutions. Even the elements sometimes wreck the paper venture. Persistent rain, a thick fog, or, worst of all, an inopportune snowstorm, anything, in fact, which interposes between the first impulse and the act of dispatch, may rob the effort of its reward. Whatever it may have been in the past, the maintenance of our unendowed hospitals is at the present time one of the hardest and most painful tasks a man or a body of men can be set. In place of the legend which now surmounts the portals of some of our noblest institutions, we might more truthfully, if less pleasantly, describe them as "supported by never-ceasing begging."

AGRICOLA.

